# Strategies for the Remediation of Micro- and Nanoplastics from Contaminated Food and Water: Advancements and Challenges

**DOI:** 10.3390/jox15010030

**Published:** 2025-02-09

**Authors:** Manikant Tripathi, Pankaj Singh, Sukriti Pathak, Ramaswamy Manimekalai, Diksha Garg, Kavya Dashora

**Affiliations:** 1 Biotechnology Program, Dr. Rammanohar Lohia Avadh University, Ayodhya 224001, Uttar Pradesh, India; 2 ICAR-Sugarcane Breeding Institute, Coimbatore 641007, Tamil Nadu, India; 3 Department of Microbiology, DAV University, Jalandhar 144012, Punjab, India; 4 Centre for Rural Development and Technology, Indian Institute of Technology, Hauz Khas, New Delhi 110016, Delhi, India

**Keywords:** microplastic, nanoplastic, biotechnology, pollution, microbial engineering, green environment

## Abstract

Micro- and nanoplastic (MNP) pollution is a significant concern for ecosystems worldwide. The continuous generation and extensive utilization of synthetic plastics have led to the widespread contamination of water and food resources with MNPs. These pollutants originate from daily-use products and industrial waste. Remediation of such pollutants is essential to protect ecosystems and human health since these ubiquitous contaminants pose serious biological and environmental hazards by contaminating food chains, water sources, and the air. Various remediation techniques, including physical, chemical, sophisticated filtration, microbial bioremediation, and adsorption employing novel materials, provide encouraging avenues for tackling this worldwide issue. The biotechnological approaches stand out as effective, eco-friendly, and sustainable solutions for managing these toxic pollutants. However, the complexity of MNP pollution presents significant challenges in its management and regulation. Addressing these challenges requires cross-disciplinary research efforts to develop and implement more efficient, sustainable, eco-friendly, and scalable techniques for mitigating widespread MNP pollution. This review explores the various sources of micro- and nanoplastic contamination in water and food resources, their toxic impacts, remediation strategies—including advanced biotechnological approaches—and the challenges in treating these pollutants to alleviate their effects on ecosystems and human health.

## 1. Introduction

Plastics have become indispensable to human life, replacing traditional materials such as wood and metal due to their cost-effectiveness and versatility. The world’s first artificial plastic was developed by Leo Baekeland in 1907 [1]. According to the International Union of Pure and Applied Chemistry (IUPAC), plastic is defined as a polymeric material that may contain additional substances to enhance performance and/or reduce costs [2]. Plastics have been described as “a material with various uses” in diverse fields, including transportation, electronics, automobiles, packaging, entertainment, and medicine [3]. But they have negative biological impacts in invertebrates, especially on annelids, mollusks, arthropods, fish, and amphibians, as well as on vertebrates [4]. Over the last five decades, plastic production has surged 20-fold, with an estimated 9200 million metric tons produced globally [1,5]. However, the large-scale utilization and non-biodegradability of plastics have made plastic pollution a pivotal global environmental concern [6]. This issue is exacerbated by unsustainable production, excessive exploitation, and the poor management of plastics [7]. Once introduced into the environment, plastics degrade into smaller fragments such as mesoplastics (>5 mm–25 mm), microplastics (<5 mm), and nanoplastics (<1 µm or 1000 nm) [8]. Microplastics (MPs) are plastic particles smaller than 5 mm, and through continuous fragmentation, they become nanoplastics (NPs), which are smaller than 100 nm in at least one dimension [9]. MPs and NPs, originating from plastics disposed of into the environment, are dangerous contaminants that threaten ecosystems, flora, fauna, and human health [2,10,11]. These particles pollute water bodies and terrestrial environments through various pathways [12,13]. UNESCO estimates that the oceans contain ~50–75 trillion fragments of plastics and MPs [14]. Even remote regions like the Tibetan Plateau report MNP contamination in rivers and lakes [15]. The presence of MNPs has also been observed in drinking water, tap water, and raw water, demonstrating their widespread distribution [16]. The persistent increase in plastic-based commodities has facilitated the entry of MNPs into food products through plastic packaging [17]. MNPs have been detected in marine environments, agricultural lands, seafood, sugar, beer, sea salt, and potable water, indicating their integration into the food web [18,19].

Micro- and nanoplastics pose severe ecological and health risks. In aquatic systems, they can be ingested by species, block digestive tracts, and cause mortality in marine organisms [20,21]. Human exposure to MNPs can result in abnormal organ development, immune dysfunction, tumorigenicity, and inflammation [22]. Additionally, MNPs impact soil characteristics, hinder plant growth, and disrupt critical soil ecosystem functions [23]. Addressing MNP pollution has become a vital area of investigation. Remediation strategies, including physical, chemical, biological, and advanced technologies like nanoremediation, are being explored to mitigate MNP pollution. This review examines the multifaceted challenges posed by MNP pollution, specifically in water and food. The manuscript holistically provides novelty by combining scientific reports on MNP contamination in food and aquatic systems, designing a multi-disciplinary comprehensive study by assessing the interaction between sources, health effects, and remediation techniques. This review highlights recent developments and cutting-edge cleanup technologies while offering suggestions for future paths of investigation. It also discusses valuable suggestions and strategies to lower MNP pollution, along with diverse sources contributing to MNP pollution, the health complications arising from these pollutants, and the recent strategies for attenuating pollution. Furthermore, this review discusses the challenges in mitigating MNP pollution, providing a comprehensive assessment of current research and future directions.

## 2. Sources of Micro- and Nanoplastics

Approximately 80% of micro- and nanoplastics (MNPs) originate from terrestrial sources, while 20% stem from aquatic sources [24]. The majority of aquatic MNP pollution is derived from terrestrial ecosystems [25]. It is estimated that approximately 800 million tons of plastics currently present in the ocean originated from land-based sources [26]. According to the United Nations Environment Program, of the 275 million tons of plastic generated in 2010, an estimated 4.8–12.7 million tons leaked into aquatic systems [27].

Based on their sources, MNPs can be categorized into two groups: primary MNPs and secondary MNPs [16]. Primary MNPs are intentionally manufactured in micro or nano sizes for various industrial applications and can directly enter into the ecosystem [28]. For instance, personal care products like cleansers, nail polish, toothpaste, and makeup products contain microbeads, which are primary MNPs. After use, these microbeads are washed into wastewater treatment plants or directly into the environment [29,30]. Primary MNPs are also used in electronic devices, automobiles, medicines, and airplanes. Secondary MNPs are produced from the fragmentation of larger plastic materials due to physical, chemical, or biological stressors such as photocatalysis, mechanical forces, oxidation, biological activity, thermal stress, and hydrolysis [16,31]. Macroplastics are converted into secondary MNPs by these degradation processes, which is the main cause of MNP pollution in aquatic environments [32]. It is difficult to measure, assess, and manage the amount of secondary MNPs that enter the oceans because their rate of production and abundance vary depending on the type of polymer and the surrounding conditions [33]. Both primary and secondary MNPs eventually infiltrate the environment, impacting soil, air, and water resources [34]. Addressing the challenges posed by these pollutants requires innovative and effective strategies to mitigate their environmental impact.

### 2.1. Terrestrial Ecosystem

As plastics are primarily manufactured, utilized, and disposed of on land, the majority of plastic waste accumulates in terrestrial ecosystems [35]. A significant proportion of micro- and nanoplastics (MNPs) in terrestrial ecosystems originate from the landfill dumping of plastic waste [16]. Approximately 49% of global plastic waste is deposited in landfills. In these landfills, the interaction of plastics with microbes, heat, moisture, and other physical conditions facilitates their degradation into MNPs. After that, these MNPs are moved in to aquatic habitats [16,36]. MNPs can also enter terrestrial ecosystems through various other pathways, including sewage sludge, agricultural practices, and uncollected plastic waste [16]. For example, in sewage sludge, industrial, domestic wastewater, landfill leachate, and urban effluents contribute to the accumulation of MNPs, with approximately 90% of MNPs being retained within it [37,38]. In agricultural practices, farming activities significantly contribute to the MNPs in soil through the use of fertilizers, pesticides, plastic mulch films, drip irrigation systems, and plastic irrigation pipes. Additionally, as greenhouse plastic covers deteriorate, MNPs are discharged into agricultural soils [39,40,41]. These sources highlight the pervasive nature of MNP pollution in terrestrial ecosystems and its potential to spread across environmental compartments. Addressing these issues requires robust waste management strategies and sustainable agricultural practices to reduce the release and negative impact of MNPs on ecosystems.

### 2.2. Aquatic Ecosystem

The major pathways for the entry of micro- and nanoplastics (MNPs) into aquatic ecosystems include water runoff, the leakage or discharge of industrial and municipal waste, fishing activities, and plastic debris floating and accumulating in water bodies [16]. The main way in which microplastics enter aquatic habitats is via urban storm water runoff as a primary pathway for microplastic pollution [42]. Similarly, Ross et al. (2023) documented the release of approximately 1.9 million to 9.6 billion microplastic particles into aquatic ecosystems during each rain event, emphasizing the critical role of urban water runoff in contributing to MNP pollution. Effective mitigation strategies are essential to address these pathways and reduce MNP contamination in aquatic ecosystems.

### 2.3. Atmosphere

The major sources of airborne micro- and nanoplastics (MNPs) include manufactured textiles, plastic erosion, cosmetics, industrial activities, and plastic manufacturing [16]. Among these, manufactured textiles, particularly synthetic fibers used in clothing, are significant contributors to atmospheric MNP pollution [43]. Synthetic clothing releases approximately 1900 fibers per wash into the environment, demonstrating its substantial role in increasing MNP concentrations in both indoor and outdoor environments [44,45]. Indoor concentrations of microplastics are generally higher than outdoor levels, likely due to confined spaces and limited air circulation [12,46]. Airborne MNPs not only contribute to atmospheric pollution but also settle on terrestrial and aquatic ecosystems, exacerbating environmental contamination [47]. Addressing airborne MNP pollution requires targeted interventions, including advancements in textile manufacturing and waste management practices, to mitigate their release into the environment.

### 2.4. Food and Water

The presence of micro- and nanoplastics (MNPs) has recently been reported across various ecological matrices, including aquatic ecosystems, the atmosphere, and terrestrial environments, suggesting their potential entry into the food web [19]. MNPs can infiltrate the human food chain through soil–plant systems [48]. Numerous food items have been found to be contaminated with MNPs, including meat, seafood, beverages, vegetables, and condiments [49,50]. It is estimated that humans consume approximately 5 g of plastic particles per person per week through food, highlighting the significant scale of MNP exposure [51]. The pervasive presence of MNPs in food underscores the urgent need for research and interventions to mitigate their impact on human health and the environment.

#### 2.4.1. Food

The extensive use of plastics and their widespread environmental prevalence have led to the significant contamination of micro- and nanoplastics (MNPs) in the food chain, resulting in human exposure. Various studies have documented the presence of microplastics in food products such as sea salt, sugar, honey, and seafood [18]. MNPs can enter food through multiple pathways. Plastics are widely used for food packaging due to their benefits, such as food preservation and ease of transportation. However, recent studies highlight the release of plastic particles from food contact materials, particularly food packaging. Researchers reported the release of MNPs from tea bags, ice cube bags, and rice bags; microplastics in packaged food ice cubes, identifying plastic packaging as a significant contamination source; and the release of MNPs from non-stick cookware, noting that the type, quality, and usage style of cookware influence contamination levels, making cookware another potential source of MNPs in food [52].

Environmental contamination is another major cause of MNP presence in plant-based foods. MNPs can infiltrate plants through root uptake, facilitated by endocytosis or root cracks, leading to contamination in fruits, vegetables, and other crops [53]. Researchers reported MNP contamination in vegetables (e.g., carrot, lettuce, broccoli, potato) and fruits (e.g., apple, pear), with apples showing the highest contamination levels [16]. This highlights the role of contaminated soil in MNP uptake during crop production and harvesting.

These findings underscore the pervasive nature of MNP contamination in both packaged and plant-derived food products, emphasizing the urgent need for interventions to minimize MNP exposure and its potential health risks.

The presence of plastic particulates has been reported in various fish and seafood products, including tuna, shrimp, prawns, crabs, sardines, and mussels [19]. This highlights that micro- and nanoplastics (MNPs) in aquatic ecosystems can contaminate food derived from water systems through aquatic organisms. Ingestion of microplastics was first examined in 220 aquatic species, including fish such as common carp, herring, Atlantic cod, and anchovies, as well as oysters and mussels [54]. These findings demonstrate the indirect infiltration of MNPs into foods sourced from aquatic ecosystems. Scientists reported microplastics in mussels sold in Cape Town, South Africa [51]. Investigators detected microplastics in the soft tissues of Indian edible mussels (*Perna perna* and *Perna viridis*) [51]. Researchers identified microplastics (<3 µm) in edible fish and mussels from the South Mediterranean coasts, emphasizing the transfer of plastic particulates from aquatic ecosystems to food resources [55]. Some of the other sources that could be potentially responsible for MNP pollution in foods are MNP-contaminated salt, sugar, and honeybees. It has been proved that honeybees are able to collect microplastics from the atmosphere, water, plants, and soil. The fibers and fragments could potentially adhere to worker bees and contaminate honey [56]. The contaminated salt and sugar used in food could also be the possible source of MNPs in food products. Researchers reported the presence of microplastics in Australian commercial salts. Microplastics were also reported in 10 Indian commercial sea salts [57]. The presence of plastic particulates has been reported by scientists in sugar samples from different supermarkets of Dhaka (Bangladesh) [57], therefore demonstrating that salt or sugar, along with honey, could significantly contribute to MNP contamination in food items.

#### 2.4.2. Water

Annually, millions of tons of plastic waste are produced, leading to the accumulation of microplastics (MPs) and nanoplastics (NPs) in freshwater systems [58]. There are multiple sources of micro- and nanoplastic contamination in water. Various researchers have reported the presence of micro- and nanoplastics in bottled water. Researchers investigated and found the presence of MNPs in bottled water packaged in plastics [59]. The concentration of microplastics was found to be lower in tap water than in bottled water [60], highlighting that the major sources of MNP contamination in water could be plastic used in water bottles [61] or potentially plastic filters used in purifying water for bottling.

Certain microplastics, as plastic particulates, can be carried by agricultural runoff into water bodies [62]. Soil erosion due to agricultural runoff exacerbates the contamination of rivers with MNPs. Additionally, scientists observed high concentrations of microplastics in agricultural runoff from biosolid-amended croplands, particularly after rainfall, suggesting that agricultural runoff is a significant non-point source of microplastic pollution in adjacent water bodies [63]. These studies emphasize the extensive and pervasive nature of MNP pollution in freshwater systems, with significant contributions from the plastic packaging of bottled water and agricultural runoff.

WWTPs (Waste Water Treatment Plants) have been considered one of the major routes for the introduction of micro- and nanoplastics (MNPs) into water bodies, as large quantities of effluent are continuously released into the water [64]. The wastewater treatment process may not remove MNPs completely [44,65,66], making WWTPs a potential source of contamination in aquatic ecosystems. According to studies, more than 45 different types of microplastic polymers have been reported in WWTPs, primarily originating from textile industries, food packaging, personal care products, and medical devices [67]. This indicates that if wastewater treatment plants are not efficient, they can release significant amounts of MNPs into the aquatic ecosystem, thereby polluting water bodies [64].

**Figure 1:**
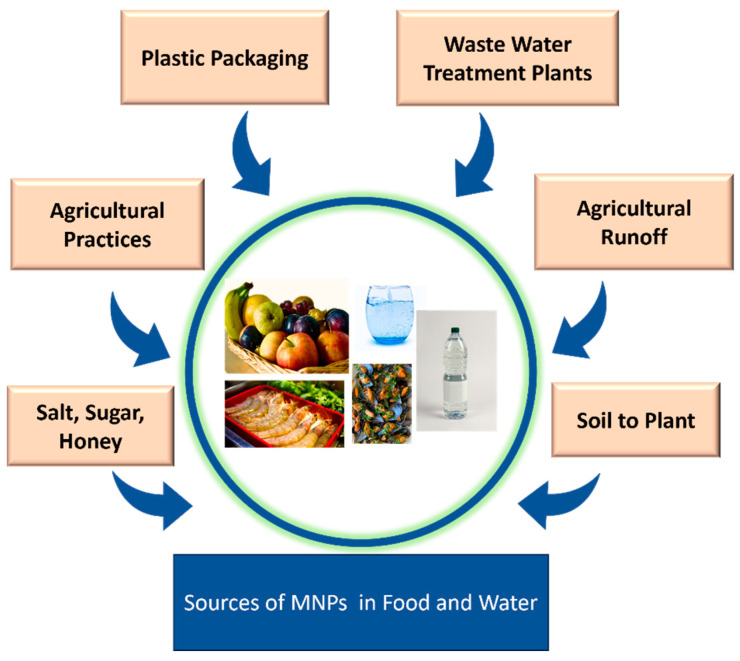
Different sources of (micro- and nano)plastics.

Another source potentially responsible for MNP pollution in water is leaching or penetration. The contamination of groundwater can occur due to the leaching or penetration of micro- and nanoplastics from the soil [68]. Various agricultural activities, waste dumping, and the use of untreated wastewater for irrigation purposes may contribute to the transport of microplastics to groundwater via the unsaturated zone, thus contaminating water sources [69]. Different sources of MNP pollution are shown in Figure 1.

## 3. Toxicity of Micro- and Nanoplastics

Plastic particulates have been proven to be hazardous to biodiversity on Earth through multiple physical, chemical, and biological impacts [70]. The toxic effects of micro- and nanoplastics (MNPs) are primarily dependent on their particle size, type, and exposure [71]. The uptake and internalization of MNPs in living organisms can occur through various pathways in plants, animals, and humans [16]. Although several toxicological effects of MNPs are attributed to their higher surface area and smaller size, other factors—such as particle shape, exposure time, functional groups, surface charge, and the type of polymer—also influence the toxicity of micro- and nanoplastics [13]. MNPs exhibit diverse toxic effects on various organisms and the environment. Aged microplastics or nanoplastics produced from degraded microplastics are more easily ingested by organisms. Once ingested, these MNPs can be absorbed into organisms’ systems, internalized, and translocated throughout the body, ultimately leading to toxicity [72]. The toxicity of micro- and nanoplastics could result not only from the particles themselves but also from pollutants adsorbed onto the micro- and nanoplastic particles [73,74].

### 3.1. Toxicity on Aquatic Ecosystem

Being the largest sink for micro- and nanoplastics (MNPs), it is crucial to understand their effects on the aquatic environment. Plastic particulates are transported and ingested by aquatic organisms [55], and the ecological invasion and accumulation of MNPs pose a significant risk to entire ecosystems [75]. Microalgae, which serve as the foundation of the aquatic food web, have been reported to be affected by micro- and nanoplastics. The toxic effects of MNPs can cause developmental toxicity, genotoxicity, or reproductive toxicity. However, these toxic effects can potentially be altered by the formation of an ecological corona [76,77,78].

**Figure 2:**
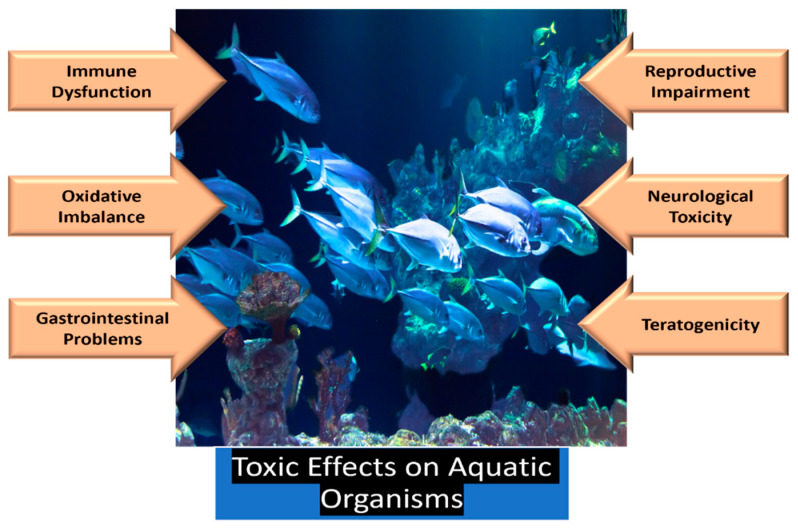
Toxic effects of micro- and nanoplastics on aquatic organisms.

Due to their small size, MNPs can be easily ingested by aquatic organisms, exerting toxicological impacts by being distributed across various tissues inside the body [13]. Several toxicological effects have been observed, including compromised immune responses, impaired neural functioning, and tissue damage. Other biological effects, such as oxidative imbalance and disturbances in energy metabolism, have also been reported [79]. Gastrointestinal problems, digestive tract injuries, reproductive and developmental toxicity, oxidative stress, neurotoxicity, and endocrine disorders are potential outcomes of MNP toxicity in aquatic organisms (Figure 2) [13].

For instance, researchers reported reduced secretion of estradiol and testosterone in zebrafish after treatment with polystyrene nanoplastics (PS-NPs) for 21 days, indicating potential damage to gonads [80]. Similarly, researchers observed suppressed acetylcholinesterase (AChE) activity in zebrafish and red tilapia, underscoring the hazardous effects of MNPs on aquatic organisms [81]. Scientists investigated the effects of MNPs on goldfish (*Carassius auratus*) larvae and reported that microplastics could induce liver, intestine, and gill damage, increase oxidative stress, and hinder growth, mobility, and heart rate [82]. Furthermore, nano-sized microplastics were found to penetrate muscle tissue and damage nerve fibers, demonstrating the dangerous and long-lasting toxicological effects of MNPs on aquatic life [82].

### 3.2. Toxicity on Human

**Figure 3:**
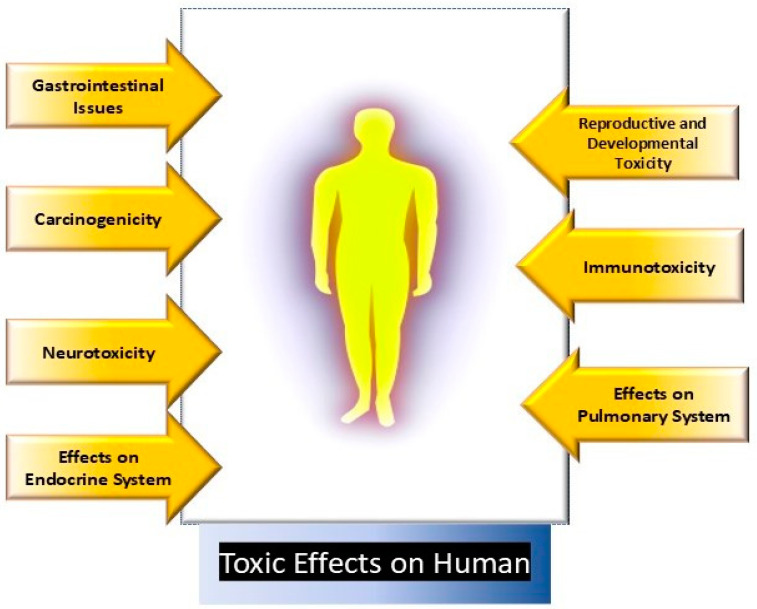
Toxic effects of micro- and nanoplastics on human.

As plastic waste continues to increase, the presence of plastic particulates, particularly microplastics (MPs) and nanoplastics (NPs), in the food chain could pose a serious threat to human health (Figure 3) [40,83,84]. The primary routes of MNP entry into the human body are ingestion (through contaminated food and water), cutaneous exposure, and inhalation (via the pulmonary system) [85,86]. MNPs can be ingested through food products like vegetables, fruits, food crops, and animal products, leading to their consumption by humans [84]. Atmospheric MNPs are commonly derived from sources such as synthetic textiles, road-wear particles, and waste incineration [87,88,89]. While skin exposure to MNPs is generally considered less significant, some reports suggest that nanoplastics can infiltrate the skin barrier [90]. Once absorbed into the body, MNPs are taken up by cells primarily via endocytosis [91]. MNPs have been detected in human blood, lungs, colon, feces, urine, sputum, spleen, breast milk, testes, placenta, and semen [85,86]. Various toxic effects of MNPs have been observed on multiple organ systems, including cytotoxicity, genotoxicity, oxidative stress, immunotoxicity, inflammation, neurological toxicity, and carcinogenicity [16]. For instance, the deposition of MNPs in the lungs could lead to fibrosis or chronic inflammation [92,93].

Several studies on cell cultures have investigated the impact of MNPs on human lung cells. Researchers reported apoptosis and inflammation caused by polystyrene nanoparticles (PS-NPs) of two different sizes (PS-NP 25 nm and PS-NP 70 nm) [94]. Researchers reported that nanoparticles affected cell viability, gene transcription, and protein expression in A549 cells [94]. Similarly, researchers reported reduced cell viability, gene alterations, inflammatory responses, and impaired lung repair in human lung epithelial cells exposed to PS-NPs, highlighting the hazardous effects of MNPs on the pulmonary system [82]. MNPs have also been detected in the human digestive system, with the presence of MNPs in feces suggesting their uptake through diet or drinking water [48]. The gastrointestinal system may be affected in various ways, including intestinal inflammation, cytotoxicity, and damage to the gut barrier, as reported by multiple researchers [95]. An inflammatory response was reported with a link to inflammatory bowel disease (IBD) when human intestinal organoids were exposed to MNPs [96]. Additionally, MNPs can promote oxidative imbalance, cellular damage, and increase the risk of neuronal disorders [97].

Microvascular toxicity caused by MNPs has also been reported, leading to thrombosis, endothelial cell damage, hemolysis, and blood coagulation [98]. While information on the effects of MNPs on the reproductive and developmental systems in humans remains limited, animal studies suggest that MNPs could cause reproductive and developmental toxicity [99,100]. Researchers reported developmental effects in the atrioventricular heart valve of human embryos and human induced pluripotent stem cells (hiPSCs) exposed to PS-NPs (40–200 nm) [101]. Scientists investigated the toxicological impacts of PS-MPs on human embryonic kidney (HEK293) and hepatocellular (HepG2) liver cells, observing significant reductions in cell proliferation, morphological changes, and increased reactive oxygen species (ROS), indicating hazardous effects on the renal and hepatic systems [102].

The reproductive toxicity of microplastics was reported in male and female mice, where the ovary size and number of follicles were reduced, reproductive hormone levels were altered, and pregnancy rates decreased. The accumulation of PS-MPs in the testes and ovaries was observed [99]. The effect of PS-MPs on neural development using a 3D model of human forebrain cortical spheroids was found to be that long-term exposure to PS-MPs altered gene expression, reduced cell viability, and suggested potential neurodevelopmental disruption [103]. Similarly, researchers assessed the effects of polypropylene microplastics (PP-MPs) on human-derived cells, observing hypersensitivity and increased ROS production, suggesting that PP-MPs at higher concentrations and smaller sizes could induce immune responses and hypersensitivity [104]. Studies on mice demonstrated the toxic effects of micro- and nanoplastics on the endocrine and skeletal muscle systems [105].

### 3.3. Toxicity on Plants

**Figure 4:**
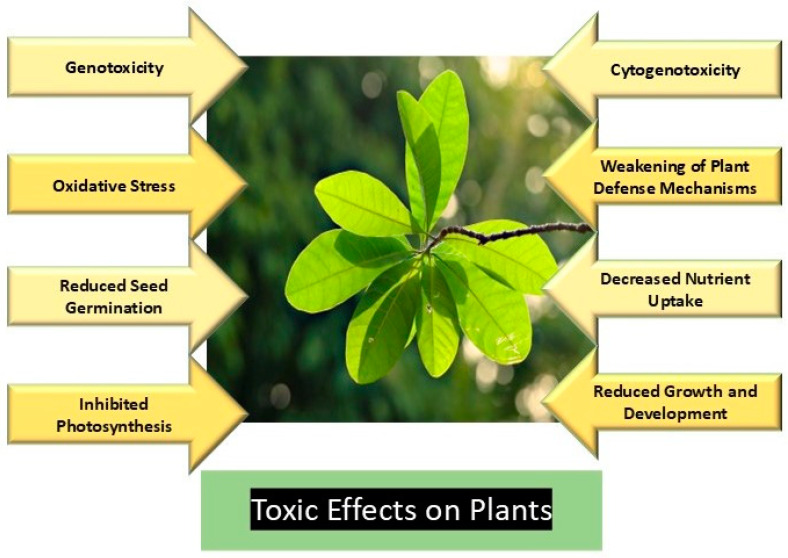
Toxic effects of micro- and nanoplastics on plants.

Oil serves as a reservoir for micro- and nanoplastics (MNPs), and the uptake and accumulation of these particles have been well documented in various plant parts such as vegetables, roots, and fruits (Figure 4) [106]. MNPs enter plants through different pathways. Nanoplastics, due to their smaller size, can be absorbed through the apoplastic pathway, while microplastics, being larger, are taken up through cracks in lateral roots or plant wounds [16,106]. MNPs can accumulate in plants through two main routes: uptake by roots and foliar absorption [73]. Smaller MNPs are more easily translocated throughout the plant, reaching the stems, fruits, and leaves. This accumulation can lead to toxic effects and poses risks to the food chain. The phytotoxicity of MNPs is influenced by their size, shape, type, and dose [46].

Research has shown that MNPs can affect various aspects of plant growth and development. For example, seed germination and early development are more adversely impacted in dicot plants compared to monocot plants [107]. Researchers investigated the effects of polystyrene microplastics (PS-MPs) and nanoplastics (PS-NPs) on common food crops, including Italian lettuce, radish, wheat, and corn, and found that the dicot plant Italian lettuce was significantly affected, showing reduced root growth and seed germination [108].

Similarly, researchers examined the short-term effects of PS-NPs on rice seedlings and observed negative impacts on seed germination, seedling growth, mitotic activity of roots, and root cell ultrastructure, along with cytogenetic aberrations [109]. Researchers studied the effects of PS-NPs on *Allium cepa* (onion) seeds and found inhibited root growth, increased oxidative stress markers, cytotoxicity, and genotoxicity [110].

Scientists assessed the impact of polyester microplastic fibers (PMFs) in a maize-based agrosystem and observed reduced crop growth, nitrogen uptake, and plant biomass production [111]. Researchers reported that microplastics damaged root cells and impaired soil nitrogen cycling, leading to reduced nitrogen uptake in peanut plants [112]. Investigators found that 100 nm micro-polystyrene particles caused morphotoxicity (reduced root length), cytotoxicity (decreased mitotic index), genotoxicity (increased chromosomal and nuclear aberrations), and downregulation of the plant’s CDKA gene, which regulates the cell cycle, further highlighting the toxicological impacts of microplastics on plants [113].

Overall, MNPs can induce a variety of detrimental effects in plants, including morphological, physiological, biochemical, metabolic, and genetic changes, as documented by several researchers [114].

## 4. Strategies for Remediation of MNPs

**Figure 5:**
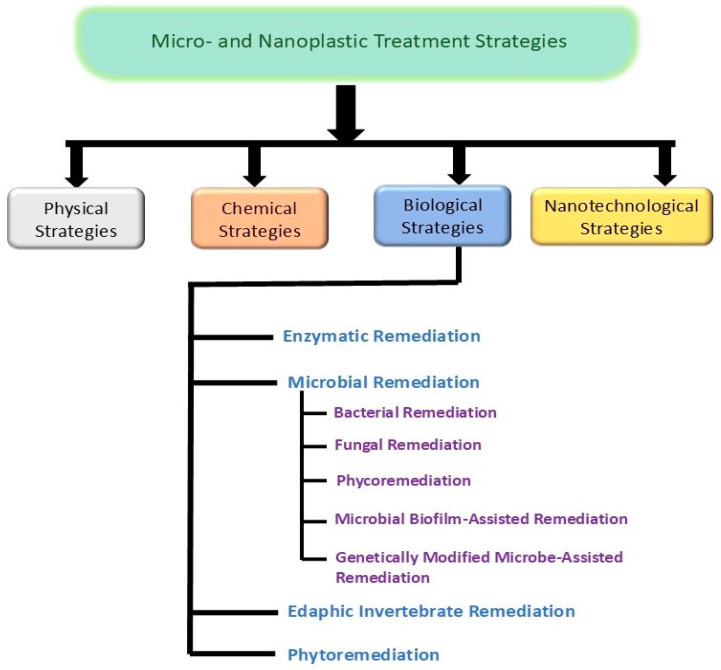
Strategies for remediation of MNPs.

Various treatment strategies (Figure 5) can be explored for the remediation of micro- and nanoplastics from the environment. Different researchers have studied diverse methods to investigate the efficient technique for the alleviation of global MNP pollution.

### 4.1. Physical Strategies

**Figure 6:**
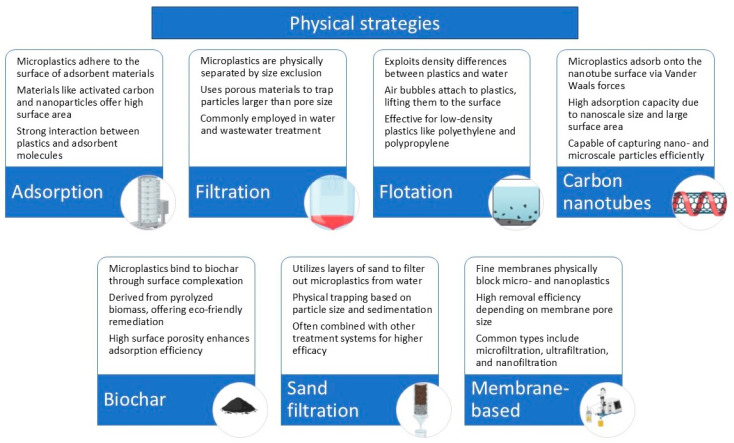
Possible mechanisms of physical strategies used for remediation of micro- and nanoplastics.

To alleviate plastic pollution, various physical treatment strategies have been developed based on the physical properties of plastics and the specific conditions of the environment. These strategies include methods such as adsorption, filtration, and flotation, which are commonly applied in wastewater treatment and plastic removal (Figure 6) [115]. The adsorption method has been widely used for wastewater treatment, particularly for removing micro- and nanoplastics (MNPs). Adsorbents like carbon nanotubes and biochar particles can immobilize or adsorb microplastics. However, while these materials can effectively capture plastics, they may also introduce new contaminants into the environment, which could complicate the overall remediation process [115,116,117,118].

Filtration methods, including sand filtration, have been explored for MNP removal. Sand filtration is a relatively simple and practical method; however, studies have shown that it has a 97% exclusion efficiency for microplastics, which means it may not fully eliminate smaller particles [119]. Additionally, membrane filtration technologies like membrane bioreactors (MBRs) have shown promise, with efficiencies of up to 99.4% for microplastic removal. Despite this, a significant drawback is membrane fouling, which is caused by microplastic accumulation. Membrane fouling can lead to irreversible damage, reducing the overall effectiveness of the technology in long-term applications [106,120].

Flotation is another physical technique used to separate plastics based on differences in density. However, this method is generally less explored for MNPs compared to adsorption and filtration, and its efficiency can vary depending on the type of plastic and environmental conditions.

It has been reported that membrane bioreactors (MBRs) can remove up to 97% nanoplastics, such as polystyrene nanoparticles (PS-NPs) from various sources [121]. However, the performance of MBRs can be negatively impacted by the presence of nano-sized particles, which increase transmembrane pressure and lead to fouling, making the system less effective over time.

In conclusion, while physical remediation techniques like adsorption, filtration, and flotation show promise for plastic pollution control, they face limitations in terms of scalability, cost, and long-term sustainability. These methods are often not sufficient on their own and may need to be combined with other approaches to achieve effective, sustainable plastic remediation in environmental settings.

### 4.2. Chemical Strategies

**Figure 7:**
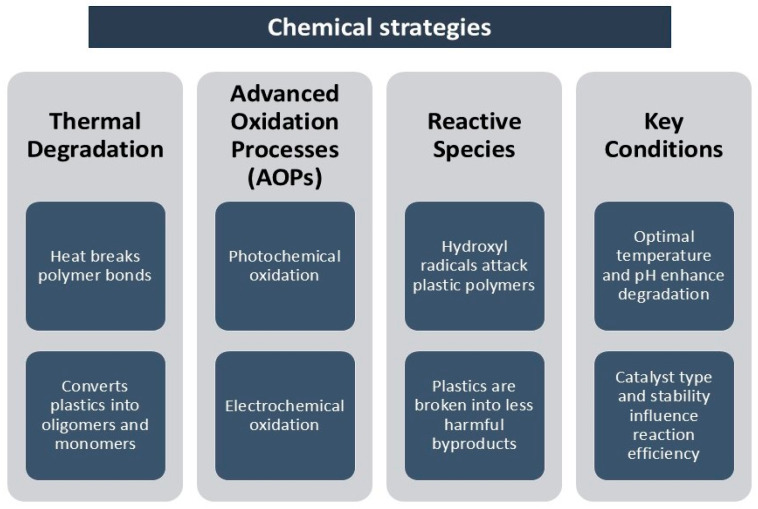
Possible mechanisms of chemical strategies used for remediation of micro- and nanoplastics.

For the chemical remediation of plastics, common degradation methods include thermal and oxidation degradation. In thermal degradation, polymers are broken down into oligomers and monomers through the uptake of heat, which leads to their disintegration [55]. Advanced oxidation processes (AOPs), which include photochemical and electrochemical oxidation, have been considered effective methods for mitigating plastic pollution [122]. These processes rely on the generation of highly reactive species, such as hydroxyl radicals, to break down plastics into less harmful substances (Figure 7).

Several factors influence the efficiency of MNP removal through AOPs, including the type and size of the micro- and nanoplastics, as well as reaction conditions such as temperature and solution pH [123]. Although AOPs are considered a promising solution for plastic pollution control, there are challenges related to the catalysts used in these processes. The development of AOP technology is still limited by the need for efficient and durable catalysts [118].

Despite their potential, AOPs face limitations due to their high energy consumption, need for specialized catalysts, and high operational costs. These factors make AOPs less sustainable and cost-effective, especially when considered for the large-scale treatment of micro- and nanoplastics [123]. Therefore, while chemical remediation methods like AOPs offer significant advantages in terms of plastic degradation, their application is still constrained by economic and technical challenges.

### 4.3. Biological Treatment Strategies

**Figure 8:**
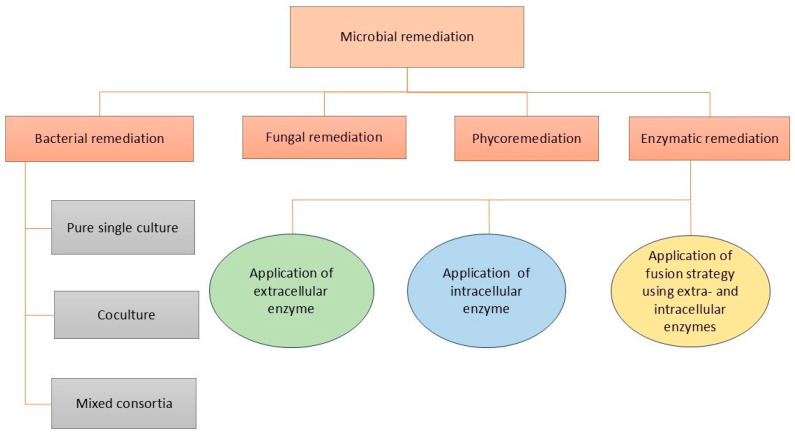
Possible microbial methods used for remediation of micro- and nanoplastics.

Microorganisms such as bacteria, algae, and fungi have been extensively investigated by researchers for their potential in the microbial remediation of micro- and nanoplastic pollution. Microbial-based methods for the remediation of these pollutants are shown in Figure 8.

#### 4.3.1. Bacterial Remediation

Microplastic-degrading bacteria have been found in various habitats, including sludge, polluted sediments, municipal landfills, and wastewater [124,125]. The enzymes involved in biodegradation include hydrolases, lipases, amidases, esterases, carboxylesterases, cutinases, and laccases [96,126,127]. Among the bacterial genera involved in the degradation of microplastics (MPs), 21% were from *Pseudomonas*, 15% from *Bacillus*, and 17% from a combination of both genera [128].

Several studies have explored the degradation of plastic particulates by bacteria. For instance, researchers investigated seven bacterial strains from plastic-polluted regions in the Mariout Wetland, Egypt, and found *Pseudomonas aeruginosa* (accession number PP087224) to be the most effective strain in degrading nanoplastics (NPs), achieving a 97% reduction in their size [129]. This highlights the potential of bacterial remediation for addressing marine nanoplastic pollution. Similarly, scientists examined the PS-MP degradation potential of *Bacillus cereus* CH6, isolated from lake sediments, and observed increased bacterial protein concentration and esterase activity related to the degradation of PS-MPs [130].

Plant growth-promoting species, *Bacillus inaquosorum* and *Bacillus velezensis*, were also found to oxidize polystyrene nanoplastics, suggesting their potential for NP degradation and the utilization of NPs for bacterial growth [131]. However, further studies are needed to enhance the efficiency and practical application of this strategy. In another study, researchers assessed the biodegradation of low-density polyethylene (LDPE) MPs by polymer-degrading bacteria, finding that *Pseudomonas aeruginosa* V1 showed the highest level of degradation, with a CO_2_ evolution of 8.86 g/L and significant weight loss [132]. Researchers examined the degradation of polypropylene MPs by marine bacterial strains from the Thoundi and Rameshwaram coasts (Tamil Nadu, India) [133]. They observed increases in bacterial growth and metabolic activity, as well as alterations in the weight, surface, and chemical composition of the PP-MPs. The potential of bacterial consortia for plastic remediation has also been studied. Researchers evaluated the potential of a bacterial consortium from plastic waste in Jakarta Bay for degrading polypropylene MPs (PP-MPs) and reported a degradation range of 2.16–6.6% (dry weight) [134]. FTIR and SEM analysis confirmed damage to the chemical bonds and surfaces of the PP-MPs, demonstrating biodegradation by the bacterial consortium. Similarly, scientists investigated the decomposition of polyethylene (PE-MPs) by mesophilic mixed bacterial cultures isolated from municipal landfill sediments, reporting efficient decomposition by *Bacillus* sp. and *Paenibacillus* sp., along with changes in chemical composition and thermal stability [135].

These studies demonstrate the potential of bacterial remediation for the sustainable and environmentally friendly management of microplastic pollution. However, further research and optimization are required to enhance the efficiency and applicability of this strategy in the future.

#### 4.3.2. Fungal Remediation

Fungi are a diverse group of organisms, primarily saprotrophs and facultative or obligate parasites. They are capable of tolerating various hazardous chemicals and metals and produce a wide array of extracellular enzymes and biological surfactants that can degrade complex polymers into simpler monomers. This ability facilitates the breakdown and mineralization of composite pollutants [136]. Several researchers have studied various fungi with the potential to degrade plastic particulates. For instance, scientists investigated the potential of the fungus *Aspergillus* sp. in aquatic environments for the biodegradation of microplastics. Their study revealed that the dominant species, *A. niger*, *A. flavus*, and *A. oryzae*, played a key role in the degradation of microplastics [137]. The enzymes involved in plastic degradation included laccase, lipase, esterase, urease, and peroxidase, all of which were found to be significant in the process.

Similarly, scientists assessed the biodegradation of three types of microplastics—polypropylene (PP), polyethylene (PE), and polystyrene (PS)—by the fungi *Aspergillus flavus* and *A. versicolor* [138]. The study showed that both fungi had higher degradation efficiency for PE, while they were less effective in degrading PP and PS. This demonstrated the potential of fungi in the remediation of microplastics. Researchers reported the effective degradation of polyethylene MPs by the fungal strain DL-1, identified as *A. niger*. After 30 days, the degradation rate of polyethylene MPs by *A. niger* was found to be 7.65 ± 0.92%, with enzymes playing a crucial role in the biodegradation process [139].

Further studies have explored the degradation of e-waste-derived microplastics, such as those from printed circuit boards (PCBs). Researchers studied the biodegradation of such microplastics by the fungus *Penicillium brevicompactum* and found that the microplastic mass was reduced by up to 75% within two weeks [140]. The fragmentation, reduced size, and increased brittleness of the MPs indicated the potential role of fungi from the genus *Penicillium*, especially *P. brevicompactum*, in the efficient biological degradation of microplastics from e-waste.

In conclusion, various fungal strains have shown potential in degrading plastic particulates, particularly microplastics, and could be utilized for green, cost-effective remediation strategies. However, further research is needed on the degradation of nanoplastics by fungi to fully understand and harness their potential for plastic pollution management.

#### 4.3.3. Enzymatic

The fundamental structure of plastic polymers consists of heteroatoms like nitrogen, oxygen, and sulfur, which serve as points where hydrolytic enzymes can act. These enzymes break down the polymers into smaller, simpler pieces that can then be consumed by microorganisms. Both microorganisms and eukaryotic organisms release these enzymes outside their cells, facilitating the biodegradation of plastics (Figure 8) [141]. There are two major types of plastic-degrading enzymes involved in the biodegradation process: extracellular and intracellular enzymes [142]. Extracellular enzymes, such as cellulases, proteases, lipases, and others, have gained significant attention for plastic biodegradation due to their broad range of functions, including oxidative and hydrolytic activities [143].

Several researchers have explored organisms with the potential for the enzymatic remediation of micro- and nanoplastics (MNPs). Researchers investigated the efficiency of the PET hydrolase enzyme from *Ideonella sakaiensis* in hydrolyzing various types of plastics, including polyvinyl chloride (PVC), polyurethane (PU), polymethyl methacrylate (PMMA), polyamide (PA), polyethylene terephthalate (PET), and polycarbonate (PC) [144]. Their study used an in silico method to assess the binding affinity of various plastic compounds to the enzyme. They found that PC and PET had the highest binding affinity with the enzyme, suggesting that these plastics were more likely to be effectively hydrolyzed, demonstrating the potential of PET hydrolase for plastic waste remediation.

Similarly, researchers examined the lipase enzyme produced by *Aspergillus niger* MG654699.1 using agro-industrial residue (specifically wheat bran) in plastic biodegradation through solid-state fermentation [145]. The degradation of polymer samples like polyethylene (PE), polyethylene terephthalate (PET), and polystyrene (PS) was analyzed using FTIR and SEM. The structural alterations and changes in the functional groups of the polymer samples indicated the impact of the lipase enzyme, highlighting its potential for eco-friendly plastic waste remediation.

Researchers studied the efficiency of a PET hydrolase (ICCG) developed through a fusion strategy for the degradation of PET-NMPs (polyethylene terephthalate nano/microplastics) [96]. They observed enhanced activity of PET hydrolase in the depolymerization of PET-NMPs, offering a stable and sustainable approach for managing PET-based pollutants. Overall, enzymatic remediation has significant potential for the effective treatment of plastic particulates, especially MNPs. However, further research is needed to optimize and efficiently implement this method for the large-scale remediation and management of plastic pollution.

#### 4.3.4. Phycoremediation

Microalgae, using enzymes, can be efficiently utilized for the biodegradation of polymers. The colonization and adhesion of microalgae on the surface of plastics in wastewater streams initiate the biodegradation process through the production of ligninolytic enzymes and exopolysaccharides [146]. Several studies have highlighted the potential of different algae species in the degradation of plastic particulates. For instance, researchers evaluated the impact of microplastics, primarily polyethylene (PE) and polypropylene (PP), on *Spirulina* sp. and its pigment phycocyanin production, while also investigating the algae’s ability to degrade PE and PP microplastics (MPs) [147]. FTIR analysis of PE and PP MPs after treatment with Spirulina sp. revealed the formation of various functional groups such as hydroxyl, ester, carbonyl, and primary alcohol, indicating the degradation of the microplastics. Moreover, a decrease in carbon content in the MPs further demonstrated biodegradation by the algae. However, the study also noted the negative impact of PE and PP on the algal species. Similarly, scientists assessed the efficiency of *Anabaena* and *Nostoc* in the degradation of synthetic polymers under lab-scale conditions. After nearly two months of treatment, the degradations were approximately 70% and 82%, respectively, for *Nostoc* and *Anabaena* species [148]. The degradation process led to the formation of monomers, which were further broken down into simpler components such as CO_2_, H_2_O, and H_2_, highlighting algae’s potential as efficient agents for bioremediation.

Researchers investigated the degradation potential of five marine microalgae strains on low-density polyethylene (LDPE) over a period of 45 days. *Picochlorum maculatum* showed the most effective degradation of LDPE [149]. SEM images of the LDPE treated with microalgae showed significant surface erosion, and ATR-FTIR analysis revealed the formation of new functional groups, suggesting the breakdown of the LDPE. This study emphasizes that phycoremediation could be a promising, green technique for the remediation of MNPs. While these studies demonstrate the potential of algae in the remediation of microplastics and plastics, further in-depth research is necessary for the effective degradation of MNPs on a larger scale.

#### 4.3.5. Microbial Biofilm-Based Remediation

The biological degradation of micro- and nanoplastics (MNPs) involves several key steps: (a) microbial biofilm formation, (b) biological deterioration, (c) biological fragmentation, and (d) biological assimilation, ultimately leading to mineralization [150,151,152]. The process begins with microbial biofilm formation, which can be influenced by various material properties, including roughness, free energy, hydrophobicity, and electrostatic interactions with the plastic surface [153]. The term “plastisphere” refers to the ecosystem of microbial biofilms that form on the surface of plastics, creating a complex community of microorganisms. Originally, the plastisphere concept was used to describe the microbial life on microplastics found in the North Atlantic subtropical gyre [154]. The biofilm consists of various microbes living in symbiotic relationships, where autotrophs (such as photosynthetic bacteria and algae) generate food, and heterotrophs rely on the organic matter produced by autotrophs. The firm surface of plastic particulates provides an ideal environment for microbial biofilm formation, which can sometimes include pathogenic microorganisms [155]. Once plastics enter the aquatic environment, microbial biofilm formation occurs on their surface, contributing to the degradation process [156].

The composition of microorganisms within the plastisphere is unique and diverse, with different microbial communities colonizing plastics depending on the type of plastic and environmental conditions [157]. Researchers studied the enhanced degradation of polyethylene (PE) film using a microbial consortium of *Arthrobacter* sp. and *Streptomyces* sp., both isolated from agricultural soil [158]. The study found that both strains exhibited the potential to degrade PE film, with *Streptomyces* sp. forming substantial biofilms. The consortium of these two strains was significantly more effective in enhancing PE film degradation compared to the individual strains. Similarly, scientists reported that biological enzymes with specific amino acid sequences were efficient in degrading polyurethane and polyethylene plastics [159]. For polyethylene terephthalate (PET) degradation, biofilm-forming microbes are predominantly from the phylum Actinobacteria, and the enzymes involved in plastic degradation belong to the group of serine hydrolases.

Microbial biofilm formation plays a critical role in the biological degradation of MNPs, and while some research has provided insights into the microbial consortia and enzymes involved, further studies are necessary to optimize and enhance the effectiveness of biological remediation of MNPs.

#### 4.3.6. Genetically Modified Microorganisms

Biotechnological approaches such as strain engineering, metagenomics, enzyme engineering, and in silico genome mining offer promising strategies for addressing micro- and nanoplastic (MNP) pollution [160]. The biodegradation of plastic particulates, particularly MNPs, by genetically engineered microbes has been explored in several studies, demonstrating the potential of these techniques to enhance the breakdown of plastics. Polyethylene terephthalate (PET), a widely used thermoplastic polymer in packaging and textiles, is known to degrade into microplastics over time. Similarly, researchers studied the degradation of PET microplastics in seawater conditions using engineered *Vibrio natriegens*, a halophilic bacterium. This system utilized a two-enzyme approach, combining IsPETase and IsMHETase from *Ideonella sakaiensis*. The engineered *V. natriegens* efficiently degraded PET, though the study highlighted several limitations that require further research to overcome.

Scientists employed genetically engineered *Escherichia coli* to display PETase on bacterial curli, creating a biocatalyst known as BIND-PETase [161]. This biocatalyst was tested for the biodegradation of PET microplastics in wastewater and the depolymerization of post-consumer PET waste. The study observed the significant degradation of PET, with the generation of degradation products at concentrations above 3000 µM under various conditions. Researchers also demonstrated the successful expression of cutinase1 (MRCUT1) from the phytopathogenic fungus *Monilio phthoraroreri* in *E. coli* [162]. This genetically engineered bacterium exhibited enhanced activity in degrading synthetic polyesters, with 59% degradation of polyethylene succinate (PES), 43% of polycaprolactone (PCL), and 31% of PET within 21 days. Researchers bioengineered *Comamonastestosteroni* CNB-1 to secrete the DuraPETase enzyme, which exhibited increased PET hydrolytic activity at room temperature. The genetically modified *C. testosteroni* CNB-1 was able to efficiently hydrolyze PET microplastics and utilize the degradation intermediates for growth, indicating its potential for the complete mineralization of PET, rather than merely breaking it down into smaller molecules [163]. Overall, these studies illustrate the significant potential of biotechnological techniques, particularly through genetically engineered microorganisms, for the efficient treatment of MNP pollution. However, there are still challenges to address, such as overcoming the limitations of current systems and expanding research to include the treatment of nanoplastics through genetically modified microbes. Further studies are required to optimize these approaches and tackle existing shortcomings for large-scale implementation.

#### 4.3.7. Edaphic Invertebrate-Assisted Remediation

The increasing presence of micro- and nanoplastics (MNPs) in soil has led to greater interactions between these pollutants and terrestrial fauna, such as slugs, snails, waxworms, grubs, and earthworms. Studies have highlighted the significant role of soil animals and their enteric microbiota in the degradation of microplastics [164,165,166]. Several investigations have demonstrated the plastic degradation potential of invertebrate soil animals. Researchers examined the biodegradation and disintegration of expanded polystyrene foam (PS) by the ubiquitous soil invertebrate *Achatina fulica* [167]. The study observed that the ingestion and degradation of PS plastics by *A. fulica* effectively contributed to plastic breakdown, demonstrating the potential of soil animals for plastic remediation. Similarly, researchers investigated the capability of earthworms (*Lumbricus terrestris*) in degrading microplastics, including low-density polyethylene (LDPE), polylactic acid (PLA), and polybutylene adipate terephthalate (PBAT) [168]. The study found no mortality in earthworms exposed to 1% MP-contaminated soil for 35 days and noted increased fragmentation of LDPE, PLA, and PBAT in the gut, suggesting that earthworms play a role in reducing MP contamination.

Additionally, research comparing the polystyrene degradation abilities of two mealworm larvae species, yellow mealworm (*Tenebrio molitor*) and dark mealworm (*Tenebrio obscurus*), found both species capable of degrading PS with a 26.03% weight loss. However, *T. obscurus* exhibited a higher degradation rate. The study further highlighted the importance of the gut microbiome in the biodegradation process of plastic particulates [169], indicating the potential of mealworms in mitigating plastic pollution. Researchers studied the biodegradation of polystyrene nanoplastics by five bacterial strains isolated from the intestinal microbiome of *T. molitor* larvae [106]. The strain *Achromobacter xylosoxidans* M9 showed significant degradation of nanoplastics, reducing the nanoparticle size by up to 92.3%.

In summary, while studies have demonstrated the effectiveness and sustainability of edaphic invertebrate-assisted remediation in treating MNP pollution, further research is needed to fill knowledge gaps and enhance the efficiency of MNP degradation by these soil organisms.

#### 4.3.8. Phytoremediation

Phytoremediation refers to the utilization of plants to remove pollutants or reduce their availability in soil. Although MNPs pose hazardous impacts on plants, various researchers have reported their potential to regulate MNP contamination [170]. Plants can decrease the concentration and/or movement of MNPs in terrestrial, aquatic, and atmospheric ecosystems through processes such as phytoaccumulation, phytostabilization, and phytofiltration [118]. Phytoaccumulation refers to the absorption and accumulation of pollutants in plants, reducing their concentration in the ecosystem. Phytostabilization involves the absorption of pollutants on roots or their precipitation in the root zone, leading to the immobilization of pollutants at contamination sites [171]. Phyto filtration refers to plants capturing MNPs by filtering them, thus preventing the uncontrolled spread of pollution [118].

Several plants have been identified for their potential in MNP phytoremediation. Scientists reported the accumulation of styrene maleic anhydride nanoparticles (NPs) in the cell walls of *Murraya exotica*, a one-year-old terrestrial plant [172]. They also observed the presence of NPs in the cytoplasm and vacuoles of the root cells. Similarly, researchers found that the aquatic floating plant *Lemna minor* could tolerate higher concentrations of microplastics (MPs) and effectively adhered to 20% of the total plastic particulates, demonstrating the phytostabilization of MNPs [173]. Another research assessed the phytofiltration potential of trees with large 3D spaces and extensive coverage [61]. These trees demonstrated the ability to intercept high-density plastics (HDPs) in the atmosphere, with an interception rate of approximately 16.3%, highlighting their potential for in situ MNP remediation.

Phytoremediation provides a sustainable and eco-friendly approach to the bioremediation of MNPs. However, various shortcomings still need to be addressed, and more research is required for the efficient and successful implementation of this strategy in the treatment of micro- and nanoplastic pollution [118].

### 4.4. Nanotechnology-Based Remediation

Nanomaterials can be utilized as flocculants, adsorbents, and catalysts in the process of remediation [174,175,176]. The magnetic extraction of microplastics from various environments using hydrophobic iron nanoparticles has proven to be efficient [177]. The integration of biological and nanoremediation techniques could provide a more effective and low-cost solution for plastic pollution [178]. However, conventional strategies for removing small plastic particles—such as physical, chemical, and biological methods—are not entirely effective in removing plastic particulates [179]. As a result, nanotechnology-based remediation strategies (Table 1) for MNP treatment have garnered significant attention in recent research. Nanomaterials substantially enhance MNP adsorption, filtration, and photodegradation [180], offering promising methods to remove MNPs from the ecosystem [181].

For example, researchers demonstrated the successful enzymatic degradation of polycaprolactone fiber mats using lipase and chitinase immobilized on nanoparticles (SiO_2_ and Fe_3_O_4_@SiO_2_), which enhanced enzymatic activity and facilitated polymer degradation [182]. Similarly, scientists investigated the degradation of polypropylene microplastics using TiO_2_ nanoparticles as photocatalysts under sunlight irradiation and observed a substantial decrease in the weight of PP-MPs, with a reduction of 50.5 ± 0.5% [183]. This suggested that the degradation of PP-MPs was supported by the generation of reactive oxygen species (ROS). Researchers also explored the photocatalytic breakdown of polystyrene (PS) microspheres and polyethylene (PE) using TiO_2_ nanoparticles under UV light irradiation [184]. They reported the efficient degradation of PS and PE, with CO_2_ as the primary end product. Complete mineralization (98.40%) of 400 nm PS microspheres was achieved within 12 h, while PE degradation achieved a higher photodegradation rate after 36 h. In a similar vein, scientists synthesized hybrid nanoparticles (humic substance/MeOxNPs) by combining the semiconductors TiO_2_ and ZnO with humic materials from biowaste biomass [185]. They assessed the antimicrobial and photocatalytic activity of these nanoparticles and observed that they exhibited increased antibacterial and antifungal capabilities, along with advanced photocatalytic activity. A 15–23% decrease in the carbonyl index of polylactic acid microplastics (PLA-MPs) was observed under UV-A and solar irradiation, indicating efficient PLA degradation.

**Table 1:** Overview of nanoparticle-assisted remediation of plastic particulates through various methods.

S. No.	Nanoparticle	Plastic Particulates	Method of Degradation	Result	Reference
1.	Graphene oxide-based metal oxide nanomaterials	Polyethylene microplastics	Photocatalytic degradation (UV light)	Mass loss of 35.66–50.46% of PE-MPs after 480 min of treatment	[186]
2.	ZnO nanoparticles (220 nm)	Polyethylene MPs (in sewage effluent)	Photocatalytic degradation	Degradation of MPs	[187]
3.	Platinum nanoparticles deposited on ZnO nanorods (ZnO-Pt) (ZnO-Pt nanocomposite photocatalysts)	LDPE film in water	Photocatalytic degradation (under visible light)	Degradation of LDPE-MP fragments	[188]
4.	Titania coated ZnOtetrapods	PE-MPs (polyethylene microparticles) and polyester microfibers	Photocatalytic degradation (UV light)	Degradation of MPs, longer degradation time (~816 h) for environmental MPs	[48]
5.	CuO/Bi_2_O_3_/g-C_3_N_4_ (Copper oxide/bismuth oxide/graphitic carbon nitride) nanoparticles	PET MP films	Photocatalytic degradation (visible light)	Degradation rate of about 41.60% after 240 h under visible light	[189]
6.	MoO_3_ nanoflakes, nanobelts and MoO_3_/SWCNT (single-walled carbon nanotube) nanocomposite	Polystyrene NPs	Photo-oxidative degradation (UV light)	Nanobelts and nanocomposite degradation of PS-NPs	[58]
7.	TiO_2_ NPs	PP-MPs (polypropylene)	Photocatalytic degradation (sunlight irradiation)	Degradation of MPs	[183]
8.	SiO_2_ and Fe_3_O_4_@SiO_2_ nanoparticles	Polycaprolactone fiber mats	(Biocatalyst) enzymes immobilized on nano carriers, enzymatic decomposition (hydrolysis/oxidation)	Degradation of polycaprolactone fiber mats	[182]
9.	TiO_2_ nanoparticle film	Polystyrene microspheres and PE (typical MPs)	Photocatalytic degradation (UV light)	Degradation of MPs	[184]
10.	Hybrid nanoparticles (humic substance/MeOxNPs)	PLA-MPs	Photocatalytic degradation (UV-A solar irradiation)	Degradation of MPs	[185]

These studies collectively demonstrate that these remediation methods are an efficient, sustainable, and environmentally friendly approach to MNP pollution. However, more research is needed to expand their application to different types of nano- and microplastics and to further optimize these technologies for large-scale environmental remediation. The advantages and limitations of various strategies have been discussed in Table 2.

## 5. Challenges and Future Perspectives

Multiple and diverse challenges exist in the effective remediation of micro- and nanoplastics from the ecosystem at various levels. Firstly, the origin of plastic particulates (primarily MPs) is difficult to trace, making it challenging to determine their conversion rate from larger particles [31]. It is also noteworthy that there is a lack of reliable data to comprehend the occurrence and spread of MNPs in agroecosystems, despite the continuous increase in plastic pollution and its mismanagement [205,206]. Secondly, the absence of any standard procedure for the isolation, quantification, and characterization of MNPs in soil is a major challenge [207]. Identifying nanoplastics in environmental samples is difficult due to the intricate nature of soil and water matrices; thus, the formation of nanoplastics in the ecosystem is not yet fully understood [208]. There are significant gaps in the data related to the sources of NPs, their transport, and hazardous effects, leading to uncertainties that complicate comprehensive environmental risk evaluation [209]. For efficient ecological risk assessments, it is important to estimate the exposure levels of MNPs and understand the movement of NPs in the environment, areas of accumulation, rates of uptake, and physiological transport to determine their probability and distribution [210]. Additionally, there is insufficient knowledge about the potential negative impacts of MNPs on humans [211].

Another major challenge is assessing the potential adverse consequences on humans and the environment due to the release of chemicals from nanoplastics as they age [212]. It is also necessary to address the task of efficiently removing MNPs using only a single treatment strategy. Therefore, the implementation of combined treatment systems to overcome the shortcomings of individual strategies should be prioritized for the effective remediation of MNPs. Most advanced treatment techniques for the removal of MNPs are currently at the small laboratory scale; their viability for large-scale implementation needs to be examined for real-world applications [64]. Additionally, strong laws, regulations, and policies are crucial for reducing plastic usage at regional, national, and international levels. Public education and awareness are also required to control plastic waste. To efficiently manage and reduce MNP pollution in the environment, there is an urgent need for a legally enforceable global framework.

To tackle these challenges, the following future perspectives are necessary:

(A) The need for additional research to generate data and assess MNP presence in geographical areas with insufficient data. These data are essential for making precise future predictions and could provide insights to address the issue of plastic pollution [213].
(B) For the identification and quantification of MNPs, various combinations of analytical tools can be utilized. Raman and µ-Raman techniques have been extensively used for analyzing MPs. On the other hand, AFM combined with infrared techniques could be used for detecting smaller particles and quantifying NPs.
(C) Long-term studies are required to assess the long-term health impacts of MNPs, primarily focusing on chronic exposure and the effects of potential interactions with other contaminants [214].
(D) For the efficient removal of MNPs, sustainable and green technologies such as bioremediation or nano remediation should be extensively explored to achieve effective MNP remediation. Additionally, the integration of multiple remediation strategies (physical, chemical, biological, biotechnological, and nanotechnological) can assist in tackling the challenges of plastic remediation from ecosystems.
(E) Various sustainable materials and alternatives should be developed in place of conventional plastics to decrease the generation of MNPs. The circular economy approach, which includes recycling and reusing plastic materials, should be developed to minimize plastic waste.

Overall, to address and solve the multifaceted challenge of MNP pollution, collaboration among scientists, policy makers, industry, and the public is crucial. This will help to develop a healthier ecosystem and ensure the safety of food and water resources from plastic contamination.

## 6. Conclusions

Plastics have had a profound impact on both social and economic livelihoods, with effects that range from supportive to harmful. The global pollution of MNPs exemplifies one of the most critical negative consequences of plastic usage. The widespread contamination of MNPs in food supplies and water bodies poses an increasing threat to human health and the environment. There are numerous sources of micro- and nanoplastic contamination in our daily lives, with toxicological impacts on human health, plants, and ecosystems.

Although various treatment strategies have been developed to mitigate the effects of MNPs, the primary challenge remains to develop an efficient, cost-effective, and sustainable approach that can be scaled up for practical, real-world application. The integration of multiple strategies is essential for the successful treatment of plastic particulates. Additionally, embracing the concept of a “circular economy,” which emphasizes reducing waste and maximizing resource efficiency, presents a promising approach to managing plastic pollution.

Research gaps in the understanding of MNP behavior, effects, and remediation need to be addressed in an efficient and comprehensive manner to develop long-term, resilient solutions. In conclusion, tackling MNP pollution requires integrated efforts from multiple fields to develop and implement strategies that protect.

## Figures and Tables

**Table 2:** Comparison of advantages and limitations of various remediation strategies.

Strategy	Advantages	Limitations	References
**Physical Strategies**			
**Adsorption**	Effective for immobilizing or capturing MNPs.	Adsorbents like carbon nanotubes and biochar may introduce new contaminants into the environment.	[190]
**Filtration (Sand)**	Cost-effective compared to advanced filtration techniques.	The exclusion is efficient for microplastics but inadequate for smaller particles like nanoplastics.	[191]
**Membrane Filtration**	Ability to eliminate polystyrene nanoparticles (PS-NPs), a kind of nanoplastic.	Susceptible to membrane fouling as a result of MNP buildup.	[192]
**Flotation**	Uses variations in density to separate plastics.	There has been limited research for MNP remediation.	[193]
**Chemical Strategies**			
**Thermal Degradation**	Breaks down polymers into monomers and oligomers using heat, which leads to disintegration.	High energy consumption and potential for the release of harmful gases during the process.	[194]
**Advanced Oxidation Processes (AOPs)**	Highly effective for plastic degradation by generating reactive species like hydroxyl radicals. It can complement other remediation methods to increase overall effectiveness.	High energy consumption and operational costs.	[195]
**Biological Treatment Strategies**			
**Bacterial Remediation**	Bacteria can degrade a variety of plastics, including micro- and nanoplastics (MPs and NPs). Biodegradation is environmentally friendly and sustainable. It can be applied to both marine and terrestrial environments (e.g., wetland, landfills, sediments).	The kind of plastic and the bacterial strain affect the rate of deterioration. Degradation rates are either slow or take a long time to have a noticeable impact. There is a possibility of partial breakdown, producing hazardous byproducts.	[196]
**Fungal Degradation of Plastics**	Fungi can tolerate toxic chemicals and metals, making them suitable for various environments. Enzymes like laccase, lipase, esterase, urease, and peroxidase play significant roles in plastic breakdown. Fungi are capable of producing extracellular enzymes and biological surfactants for degrading complex polymers.	There is not much information regarding the mechanisms of nanoplastic break down. Plastics may not completely mineralize into innocuous chemicals throughout the deterioration process. Slow degradation rates are possible. To improve the circumstances for fungi and accelerate the pace of disintegration, further study is required.	[197]
**Enzymatic Degradation of Plastics**	Complex plastic polymers can be broken down by enzymes into simpler, smaller molecules that microbes may consume. Enzymatic approaches provide eco-friendly, sustainable alternatives for plastic waste degradation.	The types of plastic and enzyme utilized affect the decomposition efficiency. Large-scale uses and scalability may be restricted by the high cost of producing enzymes.	[198]
**Microalgae for Plastic Degradation**	Microalgae produce ligninolytic enzymes and exopolysaccharides, initiating the biodegradation of plastics. The production of functional groups (hydroxyl, ester, carbonyl, etc.) on plastics indicates successful degradation.	Its growth can be negatively impacted by high concentrations of plastics, limiting their efficiency. Algae-based treatment may not be scalable for large-scale applications due to cost and resource limitations.	[199]
**Biofilm Formation on Plastics (Plastisphere)**	Microbial biofilms enhance the degradation process by forming on plastic surfaces, facilitating the breakdown of plastics. Plastisphere ecosystems create complex microbial communities that assist in plastic degradation.	The deterioration process is sluggish and takes a long time, particularly for bigger plastic pieces. Biofilms can be influenced by the roughness, hydrophobicity, and electrostatic properties of the plastic surface, which may limit efficiency.	[200]
**Genetically Modified Microorganisms (GMMs)**	GMMs can be engineered to degrade specific types of plastics (e.g., PET, PCL) more efficiently than wild-type strains. They can utilize degradation intermediates for microbial growth, leading to a more sustainable plastic mineralization process.	GMMs require rigorous safety assessments, including potential ecological risks, before release into environments. The genetic stability of engineered microorganisms can be unpredictable, affecting long-term performance.	[201]
**Edaphic Invertebrate Assisted Remediation**	Invertebrates’ gut microbiota plays a crucial role in degrading microplastics, offering a symbiotic mechanism for bioremediation.	The microbial communities in the gut can be influenced by diet and environment, affecting their degradation efficiency. The process is limited to terrestrial environments, and its effectiveness in aquatic ecosystems is uncertain.	[202]
**Phytoremediation**	Processes such as phytoaccumulation, phytostabilization, and phytofiltration enable plants to address pollution in various forms. Plants naturally integrate into ecosystems and can remediate pollution without significant disruption to surrounding wildlife.	Requires large areas of land or aquatic environments to be effective, making it less suitable for urban settings. The plants may only retain plastics in a superficial way, without fully degrading or neutralizing them.	[203]
**Nanotechnology-Based Remediation**	Nanomaterials like TiO_2_, ZnO, and SiO_2_ enhance photodegradation and enzymatic breakdown, accelerating plastic degradation.	The long-term environmental impacts of nanoparticles themselves are not yet fully understood, including potential toxicity to aquatic and terrestrial life	[204]

## Data Availability

Not applicable.
